# PIP4K2A as a negative regulator of PI3K in PTEN*-*deficient glioblastoma

**DOI:** 10.1084/jem.20172170

**Published:** 2019-03-21

**Authors:** Yong Jae Shin, Jason K. Sa, Yeri Lee, Donggeon Kim, Nakho Chang, Hee Jin Cho, Miseol Son, Michael Y.T. Oh, Kayoung Shin, Jin-Ku Lee, Jiwon Park, Yoon Kyung Jo, Misuk Kim, Patrick J. Paddison, Vinay Tergaonkar, Jeongwu Lee, Do-Hyun Nam

**Affiliations:** 1 Institute for Refractory Cancer Research, Samsung Medical Center, Seoul, Korea; 2 Research Institute for Future Medicine, Samsung Medical Center, Seoul, Korea; 3 Department of Neurosurgery, Samsung Medical Center, Sungkyunkwan University School of Medicine, Seoul, Korea; 4 Yuhan Research Institute, Yongin, Korea; 5 Institute for Cancer Genetics, Columbia University Medical Center, New York, NY; 6 Department of Health Sciences and Technology, Samsung Advanced Institute for Health Science and Technology, Sungkyunkwan University, Seoul, Korea; 7 Human Biology Division, Fred Hutchinson Cancer Research Center, Seattle, WA; 8 Department of Cancer Biology, Lerner Research Institute, Cleveland Clinic, Cleveland, OH; 9 Division of Cancer Cell Signaling, Institute of Molecular and Cell Biology, Singapore; 10 Department of Pathology, National University of Singapore, Singapore

## Abstract

Glioblastoma **(**GBM**)** is the most malignant brain tumor with profound genomic alterations. Tumor suppressor genes regulate multiple signaling networks that restrict cellular proliferation and present barriers to malignant transformation. While bona fide tumor suppressors such as *PTEN* and *TP53* often undergo inactivation due to mutations, there are several genes for which genomic deletion is the primary route for tumor progression. To functionally identify putative tumor suppressors in GBM, we employed in vivo RNAi screening using patient-derived xenograft models. Here, we identified PIP4K2A, whose functional role and clinical relevance remain unexplored in GBM. We discovered that PIP4K2A negatively regulates phosphoinositide 3-kinase (PI3K) signaling via p85/p110 component degradation in *PTEN*-deficient GBMs and specifically targets p85 for proteasome-mediated degradation. Overexpression of *PIP4K2A* suppressed cellular and clonogenic growth in vitro and impeded tumor growth in vivo. Our results unravel a novel tumor-suppressive role of PIP4K2A for the first time and support the feasibility of combining oncogenomics with in vivo RNAi screen.

## Abstract (precis)

Shin et al. identify PIP4K2A as a putative tumor suppressor in glioblastoma using an in vivo RNAi screen system. PIP4K2A competes with PTEN to negatively regulate PI3K signaling via p85/p110 component degradation in vitro and in vivo, and its expression is significantly down-regulated in GBM patients.

## Graphical Abstract

**Figure:**
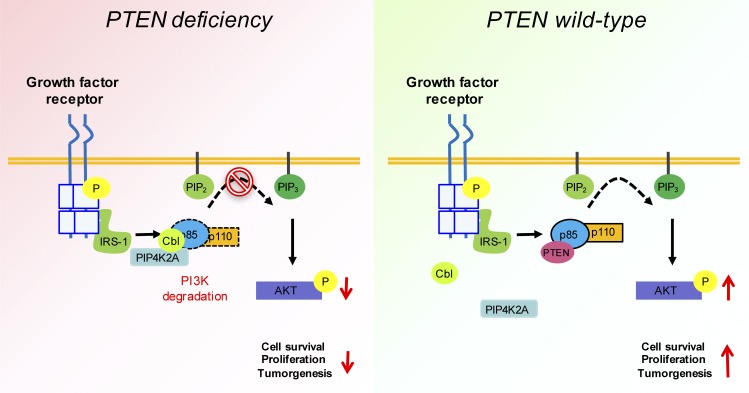

## Introduction

Glioblastoma (GBM; World Health Organization grade IV) is the most lethal primary brain tumor with standard-of-care therapies providing only partial palliation (Louis et al., 2007). Despite aggressive multimodal treatment regimens, prognosis for the vast majority of GBM patients remains dismal. GBM is the most devastating malignant form of glioma, with a median overall survival of only 14.6 mo despite the continuous progress in therapeutic interventions and innovations, including surgery, radiotherapy, photodynamic therapy, and chemotherapy (Stupp et al., 2005; Louis, 2006).

In the majority of cancer patients, including those with GBM, malignancies develop due to abnormalities in the structure and orientation of oncogenes and/or tumor suppressor genes. In part, functional loss of tumor suppressor genes is a prevalent event in tumor initiation and progression. Inactivation of tumor suppressors results from genetic alterations, including genomic mutations, allelic deletions, structure variations, as well as epigenetic silencing due to DNA methylation (Baylin, 2005). Indeed, recent large-scale genomic studies on patient-derived GBM specimens conclusively showed various somatic variances, including copy number alterations (CNAs), single nucleotide variations, fusions, exome skips, and indels (insertion/deletion; Cancer Genome Atlas Research Network, 2008; Kim et al., 2013). However, extensive genomic profiling revealed a large list of genes that may exhibit “tumor-suppressive” roles, but the frequencies of inactivating mutations are relatively uncommon, and their functional roles remain elusive (Brennan et al., 2013). Therefore, in an effort to study cancer phenotypes not readily modeled in vitro, we have adapted RNA interference (RNAi) technology to repress tumor suppressor gene functions in mice models to study aspects of tumorigenesis, tumor maintenance, and treatment response (Hemann et al., 2003; Sa et al., 2015). By implementing loss-of-function genetics in vivo model settings to closely resemble human GBM biology (Miller et al., 2017), we have identified and validated the tumor suppressive role of PIPK2A in GBM for the first time. Phosphatidylinositol signaling has been shown to impact a variety of fundamental cellular processes, including intracellular membrane trafficking, cytoskeletal rearrangement, and cell proliferation, survival, and growth. Dysregulation of these pathways could lead to malignant transformation into cancer or other diseases (Odorizzi et al., 2000; Cantley, 2002; Sasaki and Firtel, 2006; McCrea and De Camilli, 2009; Bunney and Katan, 2010; Vanhaesebroeck et al., 2012).

Phosphatase and tensin homologue (PTEN) is a tumor-suppressor protein that is often deactivated due to genomic deletion and/or mutation across a wide range of human cancers. PTEN-dependent signaling dysregulation is frequently observed in GBM, with mutation occurring in between 5% and 40% of all GBM cases and loss of heterozygosity in 60% to 80% of all cases (Srividya et al., 2011). PTEN dephosphorylates D3 position of phosphatidylinositol 3,4,5-trisphosphate (PI3,4,5P_3_), the product of activated phosphatidylinositol 3-kinase (PI3K). PI3K consists of p110 catalytic and p85α regulatory subunits that are often aberrantly activated in response to receptor tyrosine kinases. The PI3K–Akt oncogenic pathway provides proliferative and antiapoptotic signals and is frequently dysregulated in various tumor classes (Srividya et al., 2011; Grant et al., 2015). In response, PTEN functions to attenuate the PI3K–Akt oncogenic pathway and suppress its proliferative and antiapoptotic signals. In the present study, we have identified PIP4K2A as a putative tumor suppressor and demonstrated its inhibitory effects in PI3K’s signaling pathway and clonogenic growth via modulating p85/p110 PI3K complex stability in PTEN-deficient GBMs. Furthermore, we have discovered that PIP4K2A competes with PTEN for physical interaction with p85 and induces proteasome-mediated degradation, thus, demonstrating an essential tumor suppressive role in GBM.

## Results

### In vivo RNAi screen identifies putative tumor suppressors in GBM

**Figure 1.**
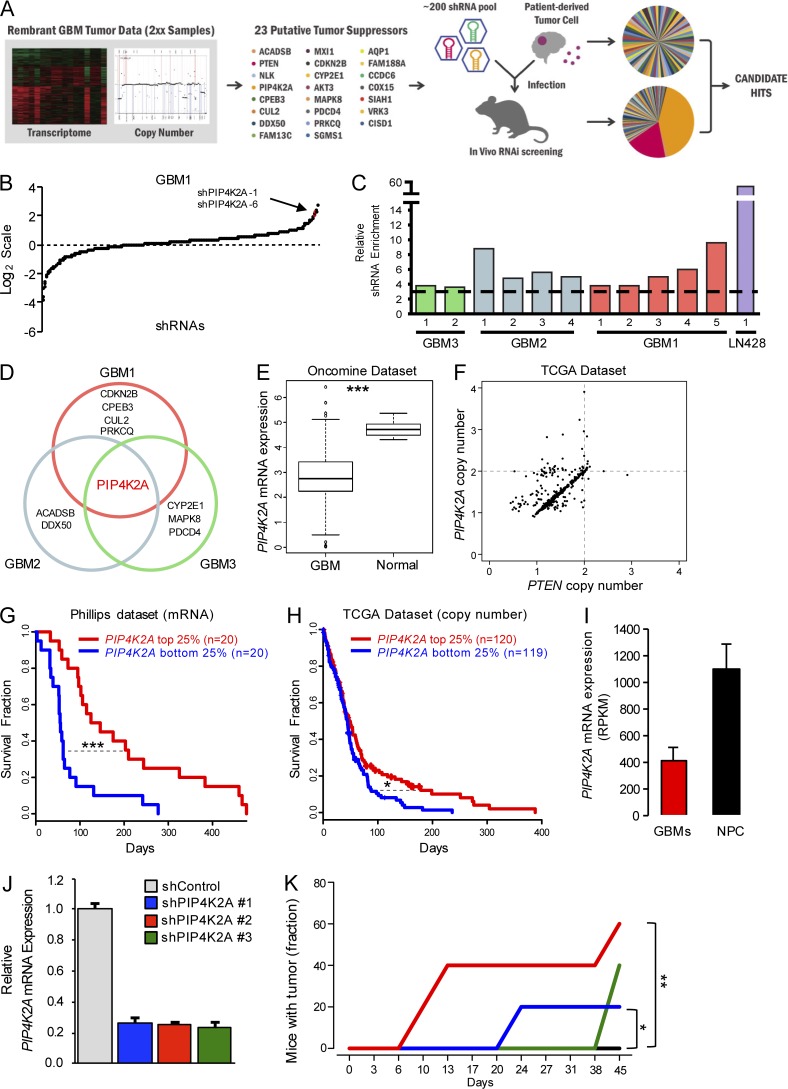
**In vivo RNAi screen identifies putative tumor suppressors in GBM. (A)** A schematic representation of in vivo RNAi screen. Candidate tumor suppressor genes were selected based on the genomic and transcriptome data from the Rembrandt database, and their target shRNA library pool was generated and transduced into patient-derived GBM cells and injected into the recipient mice brains. Harvested shRNAs were PCR amplified and deep sequenced to identify candidate “hits.” **(B)** Enrichment or depletion (log_2_ scale) of a pool of shRNAs from post–in vivo RNAi screen in GBM1. The representation of each shRNA was normalized to the initial control population. **(C)** Enrichment of PIP4K2A-targeting shRNAs from the in vivo RNAi screen. Each bar represents an individual xenograft tumor from the corresponding GBM cells. The dashed black line represents threefold enrichment compared to the control population. **(D)** Candidate hits that were enriched from the in vivo RNAi screen. **(E)** Oncomine microarray data analysis for *PIP4K2A* expression in GBM versus normal brain tissues (P = 2.129^−09^). **(F)** TCGA data analysis for *PIP4K2A* and *PTEN* copy number in GBM. **(G)** Kaplan–Meier survival curve of GBM patients based on *PIP4K2A* mRNA expression level (P = 2.95^−04^). **(H)** Kaplan–Meier survival curve of GBM patients based on *PIP4K2A* CNAs (P = 0.045). **(I)** *PIP4K2A* mRNA expression levels between sets of GBM cells and normal NPCs. RPKM stands for reads per kilobase of transcript per million reads, which derived from RNA sequencing reads. **(J)** Real-time RT-PCR analysis to determine the effects of individual shPIP4K2As on *PIP4K2A* mRNA expression level in LN-428. **(K)** Subcutaneous tumor engraftment of LN-428 cells that were infected with the corresponding indicated viruses (*n* = 5 mice per group). P values: E, two-sided Wilcoxon rank-sum test; G, H, and K, two-sided log-rank test. *, P ≤ 0.05; **, P ≤ 0.01; ***, P ≤ 0.001. Values are presented as mean ± SD.

Tumor propagation often involves particular gene “drivers” that undergo genomic alterations to initiate tumor development. Especially, loss of functional tumor suppressor genes, including *CDKN2A/B*, *PTEN*, and *RB1*, is frequently observed across a broad range of cancer types, including GBM. As genomic deletion is one of the key elements behind malignant transformation, we sought to determine putative tumor suppressor genes using genomic and transcriptome data of GBM patient specimens (*n* = 228) that are available from the Rembrandt database. The vast majority of the candidate genes were located on chromosome 10, as loss of heterozygosity on chromosome 10 is a common genetic event during GBM progression. We selected five to seven individual shRNA clones targeting each candidate gene from Cold Spring Harbor shRNA libraries and generated them in a pooled format. By integrating each tumor cell with an individual unique shRNA, we reasoned that a subset of population would outgrow the rest due to selective growth advantage. After a thorough optimization process, we generated orthotopic xenograft models that were injected with shRNA pool-transduced GBM cells (Fig. 1 A). Three different patient-derived primary GBM cells and one human GBM cell line, LN-428, were used to cover the diverse genotypes and phenotypes of GBM. After tumors were harvested, DNA was extracted, and shRNA hairpins were PCR amplified and deep sequenced for hairpin representation analysis.

Through strict criteria, we identified several candidate hits whose shRNA representations were enriched in two or more independent xenograft tumors from two or more different GBM models (Fig. 1, B–D). PIP4K2A emerged as a robust hit in all four GBM patient-derived xenograft models, and no previous reports have demonstrated its functional or clinical relevance in GBM. To assess clinical relevance of PIP4K2A, we surveyed the mRNA expression level of *PIP4K2A* in GBM tumors compared with normal brain tissues and found that the *PIP4K2A* mRNA level was significantly lower in GBM (Fig. 1 E). As both PIP4K2A and PTEN are located on chromosome 10, we cross-analyzed their copy number variations and mRNA expression levels using The Cancer Genome Atlas (TCGA) patient GBM specimens and discovered that most patients exhibited dual genomic deletions or low transcriptome levels whereas only a small subset of patients has either *PIP4K2A* or *PTEN* deletion alone (Fig. 1 F and Fig. S1, A and B). Since a large number of tumors harbor both genomic deletions of *PIP4K2A* and *PTEN*, we further verified whether sole PIP4K2A gene alteration or expression would exhibit a distinctive survival difference in GBM patients. When we stratified GBM patients according to *PIP4K2A* mRNA expression level or CNA, the *PIP4K2A^high^* group showed significantly favorable outcomes compared to the *PIP4K2A^low^* group (Fig. 1, G and H; and Fig. S1, C and D). We further analyzed the mRNA expression level of *PIP4K2A* in patient-derived GBM cells compared with the neural progenitor cells (NPCs) and found that *PIP4K2A* expression was significantly higher in NPCs compared to the GBMs as well (Fig. 1 I). To further validate our RNAi screen results, we evaluated the effect of each individual shRNA directed against PIP4K2A on the growth of GBM xenografts. Consistently, silencing of PIP4K2A by single shRNAs resulted in significantly faster tumor engraftments compared with the control group (Fig. 1, J and K).

**Figure 2.**
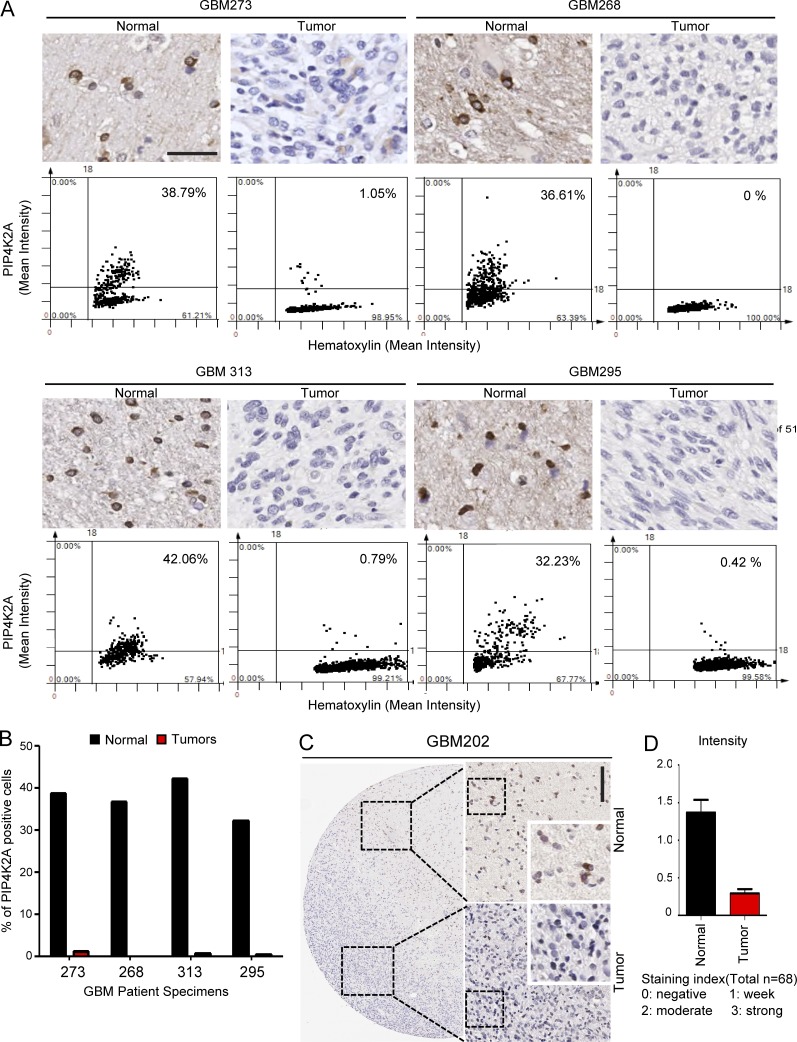
**PIP4K2A protein expressions in human GBM specimens. (A)** TMA of PIP4K2A on 88 GBM and 32 matched normal brain specimens. Quantitative assessment of PIP4K2A immunohistochemical signals in both GBM and matching non-neoplastic tissues was measured on intensity over area or intensity, respectively. Intensity was measured in different areas of the tissue that were selected randomly (±SD, *n* = 4). Bar, 100 µm. **(B)** Bar graph representation of PIP4K2A-positive cells. **(C)** Immunohistochemical analysis of PIP4K2A in GBM202 patient. Bar, 200 µm. **(D)** Bar graph representation of PIP4K2A expression intensity on normal tissue versus tumor regions.

As *PIP4K2A* is found to be highly expressed in NPCs and normal brain tissues according to the public database, we conducted TissueFAX microscopy analysis (TMA) of 88 GBM tissue specimens and their adjacent non-neoplastic tissues to determine PIP4K2A protein expression levels. Quantification of immunohistochemical analyses using the TMA system revealed significant differences between matched neoplastic and non-neoplastic tissues as PIP4K2A protein level was much higher in the non-neoplastic regions compared with its corresponding tumor sections, consistent with our previous results (Fig. 2). Collectively, our results highlight PIP4K2A’s tumor suppressive potentials within clinical framework.

### PIP4K2A attenuates cellular proliferation, clonogenic growth, and AKT signaling pathway in patient-derived GBMs

**Figure 3.**
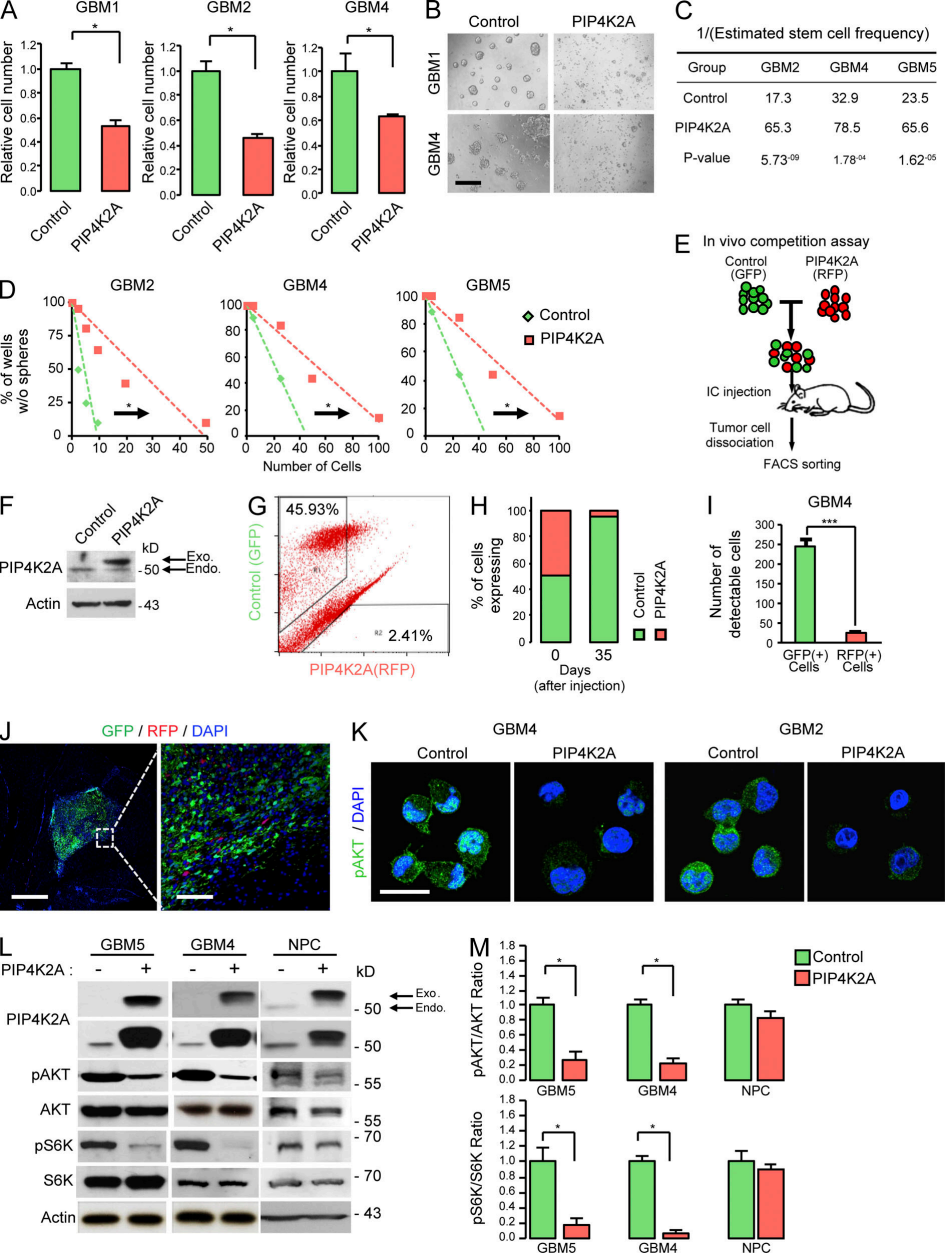
**PIP4K2A negatively regulates cellular proliferation, clonogenic growth, and the AKT signaling pathway in patient-derived primary GBMs. (A)** Effects of PIP4K2A on in vitro proliferation of GBM cells that were transduced with either control (non-target) or PIP4K2A-expressing virus. Values are presented as mean ± SD (*n* = 5). **(B)** PIP4K2A suppresses tumorsphere formation. Bar, 100 µm. **(C and D)** LDA for in vitro tumor sphere formation. LDA clonogenic significance was measured by linear regression analysis. **(E)** A schematic representation of dual-color competition assay of GBM4 cells in vivo. A total of 100,000 cells from a 1:1 mixture of RFP-labeled PIP4K2A WT cells (red) and GFP-labeled control (NT) cells (green) was injected into the mouse brains. **(F)** Immunoblot analysis of PIP4K2A in patient-derived GBM4 cells transduced with NT control or PIP4K2A-WT vector. **(G)** FACS analysis of post–in vivo dual-color competition assay. **(H)** Bar graph represents the relative percentage of GFP- and RFP-positive cells from the FACS analysis. **(I)** Representative bar graph of the number of GFP- and RFP-positive cells that were counted from different regions that were selected randomly from the post–in vivo competition assay. Values are presented as mean ± SD (*n* = 4). **(J)** Immunofluorescence images of cryosectioned mouse brains from the in vivo dual-color competition assay. Bars, 1,000 (left) and 100 µm (right). **(K)** Representative confocal images of immunofluorescence staining of pAKT in GBM cells that were transduced with NT virus or PIP4K2A-expressing virus. Bar, 20 µm. **(L)** Representative immunoblot analysis of phosphorylated-AKT (pAKT), total AKT, phosphorylated-S6K (pS6K), total S6K, and PIP4K2A expression in patient-derived tumor cells and NPCs after they were transduced with either PIP4K2A or control. Actin was used as a loading control. **(M)** Densitometric analysis of the blots in L. Values are presented as mean ± SEM. Data shown in A–D, F, and K–M are representative of three independent and reproducible experiments. P values: A and M, two-tailed Student’s *t* test. *, P ≤ 0.05; ***, P ≤ 0.001. Endo., endogenous; Exo., exogenous.

To explore the functional role of PIP4K2A in GBM, we first investigated its ability to regulate cellular proliferation. Patient-derived GBM cells were used to evaluate PIP4K2A’s potential tumor-suppressive role because they exhibit minimal *PIP4K2A* expression levels. Transduction with lentivirus expressing *PIP4K2A* wild-type (PIP4K2A) significantly inhibited cellular viability and cellular growth in PIP4K2A-transduced GBM cells compared to the control groups (Fig. 3, A and B; and Fig. S2 A), whereas shRNA-mediated knockdown of *PIP4K2A* increased the cellular growth of *PIPK2A* wild-type cells compared with the non-targeting shRNA control (Fig. S2, A–C).

Cancer stem/initiating cells possess an innate ability to initiate tumor growth in vivo. Although some cancers may not directly follow the cancer stem/initiating cell architecture, multiple studies have shown that GBMs harbor a subset of highly tumorigenic, stem-like cells called glioma stem cells (GSCs; Singh et al., 2004; Lee et al., 2006; Dirks, 2010; Sa et al., 2015). They have been frequently identified in grade IV gliomas (Singh et al., 2004; Yuan et al., 2004) and enriched with self-renewal capacity, which may contribute to the aggressive behavior of GBM (Mangiola et al., 2007; Stiles and Rowitch, 2008; Sa et al., 2015). Given a growth-inhibitory effect of PIP4K2A, we suspected that PIP4K2A could regulate stem-like properties of GBM as well.

Clonogenic growth in the form of neurospheres is an in vitro indicator of GSC self-renewal capability (Pastrana et al., 2011). To assess the effect of PIP4K2A in GSC self-renewal, we performed neurosphere formation–limiting dilution assays (LDAs). Overexpression of PIP4K2A decreased sphere-forming capability in primary GBMs compared with the control group, indicating an inhibitory effect of PIP4K2A on clonogenic growth of GSCs (Fig. 3, C and D).

To further interrogate the role of *PIP4K2A* in tumor growth in vivo, we performed in vivo growth competition assays in which control (GFP-labeled) and PIP4K2A-transduced tumor (RFP-labeled) cells were equally mixed at a 1:1 ratio and co-injected into mouse brains (Fig. 3, E and F). Once tumor formation was confirmed, we harvested the resulting tumors and performed FACS (Fig. 3, G and H) and immunofluorescence analysis (Fig. 3, I and J). In corresponding in vivo models, more than 90% of the resulting tumor cells were derived from GFP-positive, PIP4K2A-nonexpressing cells (control cells). These results strongly demonstrate that PIP4K2A impedes tumor propagation in vivo*.*

As previous results have shown that PIP4K2A could suppress GBM cellular proliferation and clonogenic growth, we further investigated downstream effectors of its pathway. PIP4K2A-transduced GBM cells exhibited significantly attenuated levels of AKT (Ser473) and S6K phosphorylation, a downstream target of AKT (Fig. 3, K–M). On the contrary, PIP4K2A overexpression showed little or no effect on NPCs (Fig. 3, L and M; and Fig. S3, A and B). Because overexpression of PIP4K2A could potentially confer different functions than its intended role, we evaluated expression level of exogenous PIP4K2A within the physiological range. When we directly compared the exogenous level of PIP4K2A in GBM that was within the physiological range of endogenous PIP4K2A level in NPCs, we discovered that overexpression of PIP4K2A consistently attenuated phosphorylation of AKT (Fig. S3 C). Collectively, our results demonstrate that PIP4K2A promotes attenuation of the AKT signaling pathway, contributing to its inhibitory effects in tumor cellular proliferation and clonogenic growth.

### PIP4K2A regulates p85 protein stability through the ubiquitin-proteasome–mediated pathway

**Figure 4.**
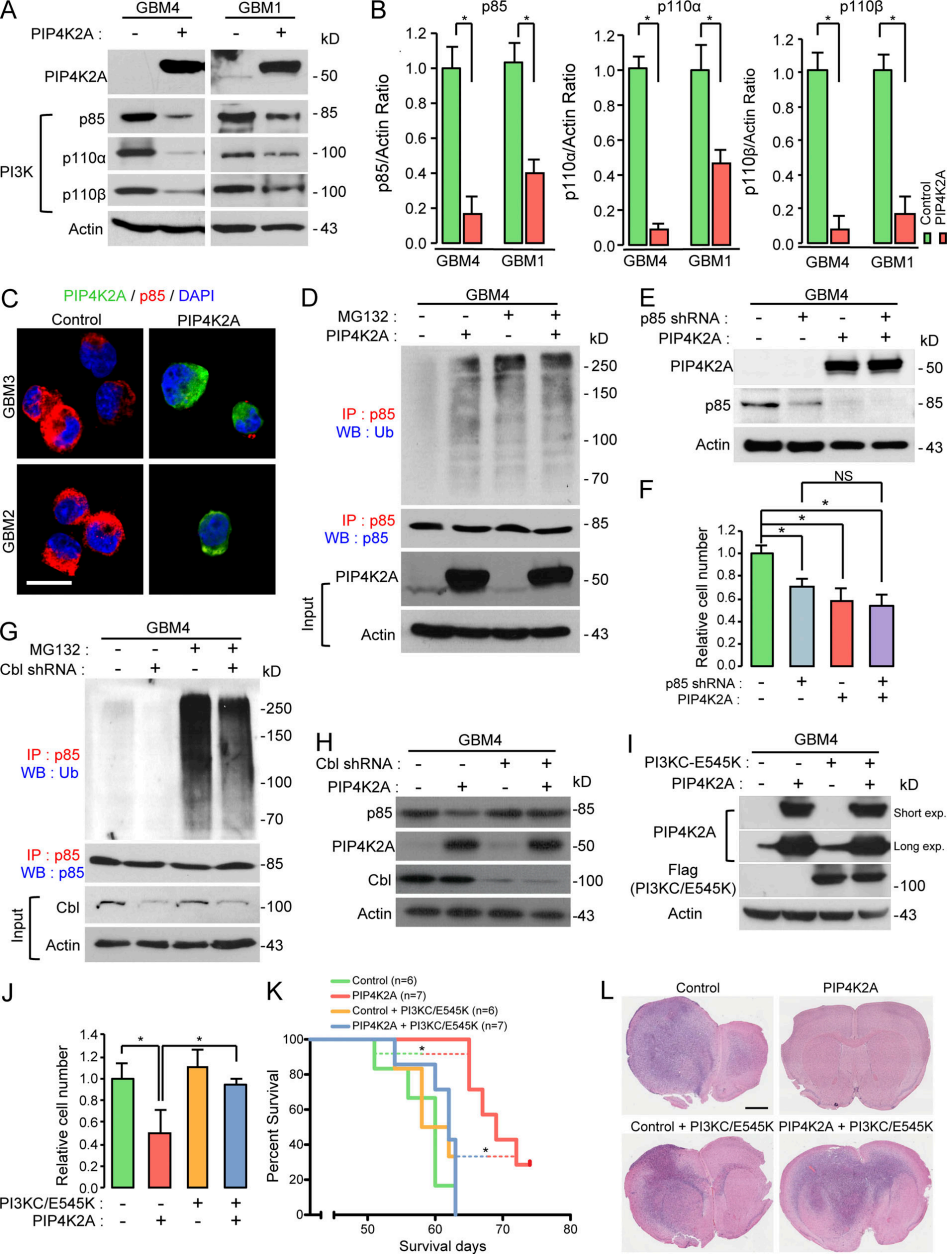
**PIP4K2A negatively regulates PI3K signaling through degradation of p85 via Cbl-p85 ubiquitination. (A)** Immunoblot analysis of PI3K complex (p85, p110α, and p110β) in patient-derived GBM cells after transduced with either control or PIP4K2A. Actin was used as a loading control. **(B)** Densitometric analysis of the blots in A. Values are presented as mean ± SEM. **(C)** Representative confocal images of immunofluorescence staining of PIP4K2A and p85. Bar, 20 µm. **(D)** Co-IP of ubiquitin and p85 in GBM cells that were transduced with either PIP4K2A or control and treated with or without MG132. For IP immunoblotting data, antibodies used for IP and Western blotting (WB) were labeled as red and blue, respectively, and p85 was used as a loading control. For evaluation of PIP4K2A overexpression in input data, actin was used as a loading control. **(E)** Immunoblot analysis of p85 and PIP4K2A in GBM cells that were transduced with control, p85 shRNA, PIP4K2A, or p85 shRNA/PIP4K2A. Actin was used as a loading control. **(F)** Proliferation assay of GBM cells from F. Values are presented as mean ± SD. **(G)** Co-IP and immunoblot analysis of p85 and ubiquitin in GBM cells that were transduced with either control or Cbl shRNA and treated with or without MG132. p85 was used as a loading control. **(H)** Immunoblot analysis of p85, PIP4K2A, and Cbl in patient-derived GBM cells that were transduced with control, PIP4K2A, Cbl shRNA, or PIP4K2A/Cbl shRNA. Actin was used as a loading control. **(I)** Immunoblot analysis of PIP4K2A and anti-Flag (PI3KC-E545K mutant) in GBM cells that were transduced with control, PIP4K2A, PI3KC-E545K, or PIP4K2A/PI3KC-E545K. Actin was used as a loading control. **(J)** Proliferation assay of GBM cells from I. Values are presented as mean ± SD. **(K)** Kaplan–Meier survival curve of mice that were orthotopically implanted with GBM cells transduced with control (*n* = 6), PIP4K2A (*n* = 7), control/PI3KC-E545K (*n* = 6), or PIP4K2A/PI3KC-E545K (*n* = 7). **(L)** Representative H&E sections of the mouse brains from K. Bar, 2 mm. Data shown in A–J are representative of three independent and reproducible experiments. P values: B, F, and J, two-tailed Student’s *t* test*;* K, two-sided log-rank test. *, P ≤ 0.05. Exp., exposure; WB, Western blot.

To identify the potential underlying mechanism by which PIP4K2A inhibits AKT signaling, we investigated its involvement in the PI3K pathway, the upstream regulator of AKT. PI3Ks are heterodimers composed of p85 regulatory and p110 catalytic subunits, of which there are several isoforms that have been reported to catalyze production of the lipid second messenger PI3,4,5P_3_ in the cell membrane. The interaction between p85 and p110 is integral to the stability of p110 (Yu et al., 1998a,b). Therefore, we evaluated the interaction between ectopic expression of PIP4K2A and PI3Ks. As suspected, PIP4K2A down-regulated basal expression levels of p85, p110α, and p110β (Fig. 4, A–C), attenuating AKT phosphorylation in process.

To explore the molecular mechanisms underlying PIP4K2A-induced p85 instability, we examined whether the diminished expression level of p85 was induced through proteasomal degradation. Interestingly, pretreatment of MG132, a proteasomal inhibitor, and PIP4K2A overexpression promoted ubiquitination-mediated proteasomal degradation of p85 (Fig. 4 D and Fig. S3 D). Moreover, we observed no additional inhibition effect in cellular growth when we performed simultaneous shRNA-mediated knockdown of p85 with PIP4K2A overexpression, further advocating that PIP4K2A suppresses tumor growth through directly down-regulating p85 (Fig. 4, E and F). Additionally, shRNA-mediated silencing of Cbl attenuated degradation of p85 in the presence of PIP4K2A (Fig. 4, G and H). Although Cbl was introduced as an E3 ubiquitin ligase for p85 in T cells, it did not exert proteasomal degradation of p85 (Fang and Liu, 2001; Ko et al., 2014). The identity of the E3 ligase that is responsible for p85 degradation and its biological significance has not been previously reported. Hence, our finding of PIP4K2A-mediated proteasomal degradation of p85 through Cbl was striking.

### PIP4K2A suppresses in vivo tumorigenesis through down-regulation of p85

To further verify whether PIP4K2A-mediated tumor impediment is dependent on down-regulation of PI3K/AKT signaling, we conducted in vitro and in vivo rescue experiments (Fig. 4, I–L). The majority of *PIK3CA* mutations occur within two “hotspots,” in the kinase (H1047R) and helical domains (E542K and E545K) of p110α (Samuels et al., 2004; Bader et al., 2005; Meyer et al., 2013). These mutations lead to constitutively active enzymes, transforming cells in vitro, and enhancing tumorigenicity in xenograft models (Isakoff et al., 2005; Zhao et al., 2005; Koren and Bentires-Alj, 2013). Notably, ectopic expression of constitutively active PI3KC mutant (E545K) restored the tumor propagation capacity of PIP4K2A-transduced GBM cells (Fig. 4, K and L). Together, our results suggest that PIP4K2A facilitates in vitro and in vivo tumor growth inhibition via down-regulating p85–*PIK3CA* axis.

**Figure 5.**
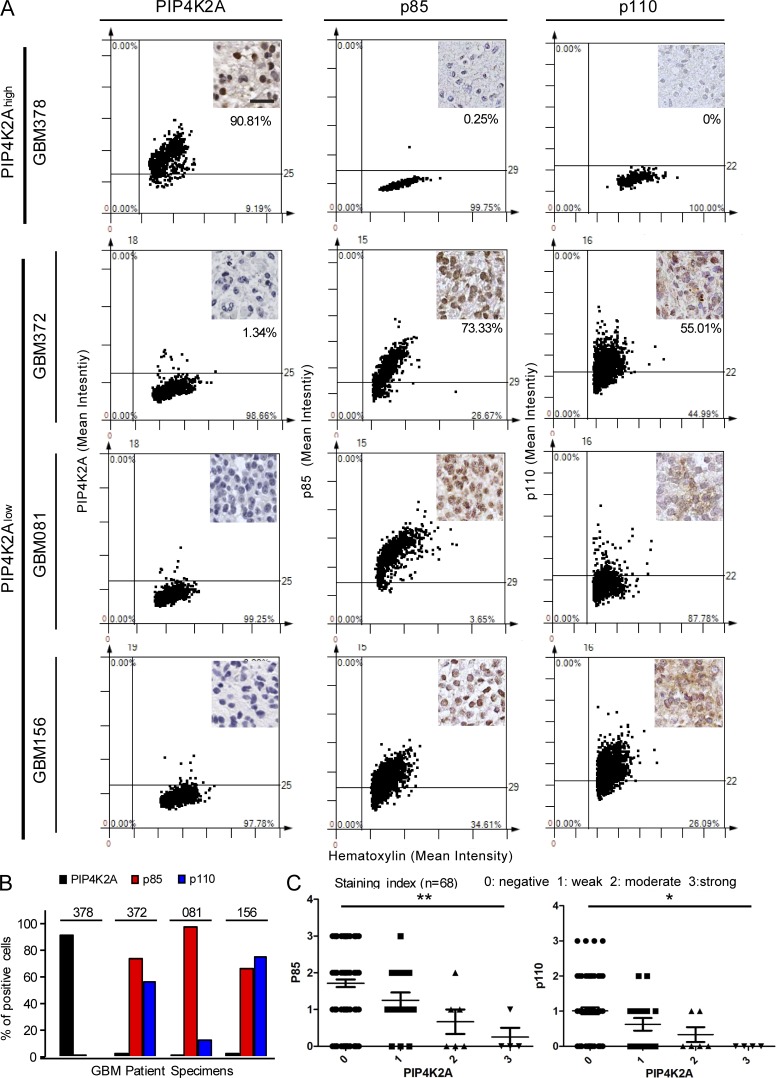
**Interrelation between PIP4K2A and PI3K subunits in human GBM specimens. (A)** Representative immunohistochemical images and TMA of PIP4K2A, p85, and p110 on 88 GBM brain specimens. Quantitative assessment of PIP4K2A, p85, and p110 immunohistochemical signals in GBM tissues was measured on intensity over area. Intensity was measured in different areas of the tissue that were selected randomly (±SD, *n* = 4). Bar, 50 µm. **(B)** Bar graph representation of PIP4K2A, p85, and p110 positive cells. **(C)** Bar graph representation of PIP4K2A expression with p85 (left) or p110 (right) expression intensity on randomly selected tumor regions. P values: C, one-way ANOVA test.

As we have previously shown that GBM tissue specimens commonly exhibited low protein expression level of PIP4K2A, we further investigated expression levels of p85 and p110 to determine the potential clinical association between PIP4K2A loss and PI3K encoding genes. PIP4K2A^high^ tumor (GBM378) exhibited minimal p85 and p110 protein expression levels, while PIP4K2A^low^ tumors (GBM156, GBM081, and GBM372) showed significantly higher levels of p85 and p110 (Fig. 5 A). Collectively, our results highlight a strong inverse correlation between PIP4K2A and p85/p110 protein levels, prompting clinical significance of PIP4K2A with p85 and p110 in GBM (Fig. 5, B and C).

### PIP4K2A and p85 protein interaction in *PTEN*-deficient GBM

**Figure 6.**
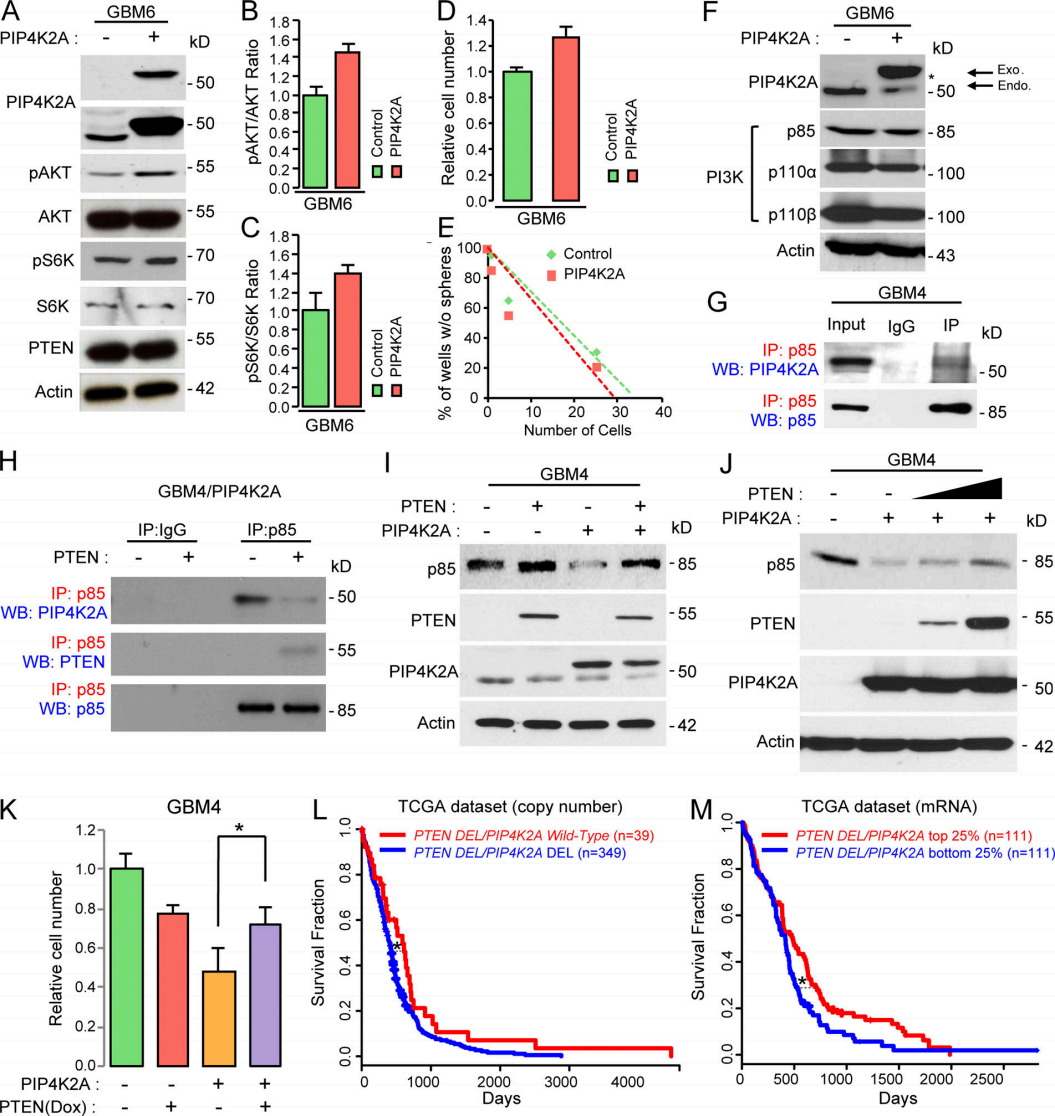
**PIP4K2A protein interacts with p85 in *PTEN*-deficient GBM. (A)** Immunoblot analysis of PIP4K2A, pAKT, AKT, pS6K, S6K, and PTEN in patient-derived GBM cells after they were transduced with either control or PIP4K2A. Actin was used as a loading control. **(B and C)** Densitometric analysis of the blots in A. Values are presented as mean ± SEM. **(D)** Effects of PIP4K2A on in vitro proliferation of GBM cells that were transduced with either control or PIP4K2A. Values are presented as mean ± SD (*n* = 5). **(E)** LDA for in vitro tumor sphere formation. LDA clonogenic significance was measured by the linear regression analysis. **(F)** Immunoblot analysis of PI3K complex (p85, p110α, and p110β) in patient-derived GBM cells that were transduced with either control or PIP4K2A. Actin was used as a loading control. **(G)** Co-IP analysis of PIP4K2A and p85 in *PTEN*-deficient GBM cells. IgG represented a control antibody used for IPs. **(H)** Co-IP analyses of p85, PIP4K2A, and PTEN in GBM cells that were transduced with either control or PTEN. IgG represented a control antibody used for IPs. **(I)** Immunoblot analysis of p85, PTEN, and PIP4K2A GBM cells that were transduced with control, PTEN, PIP4K2A, or PTEN/PIP4K2A. Actin was used as a loading control. **(J)** Immunoblot analysis of p85, PTEN, and PIP4K2A in GBM cells that were transduced with control, PIP4K2A, or doxycycline (Dox)-induced PTEN in a dose-dependent manner. Actin was used as a loading control. **(K)** Proliferation assay of GBM cells from I. Values are presented as mean ± SD. **(L and M)** Kaplan–Meier survival analysis of *PTEN*-deleted GBM patients based on *PIP4K2A* copy number (L) or mRNA expression (M) from TCGA dataset. Data shown in A–K are representative of three independent and reproducible experiments. P values: K, two-tailed Student’s *t* test; L and M, two-sided log-rank test. *, P ≤ 0.05. Endo., endogenous; Exo., exogenous; WB, Western blot.

We have previously shown that PIP4K2A significantly down-regulated the AKT/S6K pathway in *PTEN*-deficient GBMs (Fig. 3, L and M; and Fig. S2 A). However, similar observations were not speculated in a subset of GBMs with *PTEN* wild-type (Fig. 6, A–C; and Fig. S2 A). Overexpression of PIP4K2A did not suppress cellular proliferation and clonogenic growth in *PTEN* wild-type GBM cells compared with the control group (Fig. 6, D and E). Additionally, p85/p110 complex structure was not affected by PIP4K2A in *PTEN* wild-type cells, as well (Fig. 6 F).

Recent studies have shown that PTEN binds directly with p85 and enhances PTEN lipid phosphatase activity (Chagpar et al., 2010). Therefore, we suspected that PIP4K2A may physically compete with PTEN for interaction with p85. To investigate the interactive role of PTEN in PIP4K2A-mediated degradation of p85, we first conducted co-immunoprecipitation (IP) experiments. Through IP with an anti-p85 antibody followed by immunoblotting using PIP4K2A antibody, we found that PIP4K2A directly binds with p85 in *PTEN*-deficient GBM (Fig. 6 G). However, PIP4K2A-p85 interaction in *PTEN*-deficient GBM was significantly ablated after the cells were transduced with PTEN wild-type, through the inducible-doxycycline technique, demonstrated by IP immunoblot analysis (Fig. 6 H). Furthermore, ubiquitination-mediated proteasomal degradation of p85 was also reduced upon PTEN expression (Fig. S3 E). We also discovered that p85 protein expression level and cellular growth were restored in a dose-dependent manner in the presence of PTEN after PIP4K2A overexpression (Fig. 6, I–K). Notably, in *PTEN*-deletion cohorts, patients with *PIP4K2A* wild-type or transcriptional up-regulation demonstrated significantly favorable survival outcomes compared to the *PIP4K2A* deleted or down-regulated group, further advocating that tumor suppressive role of PIP4K2A depends on PTEN deficiency (Fig. 6, L and M). Collectively, our results highlight that PIP4K2A competes with PTEN for p85 interaction and subsequently mediates p85 degradation in *PTEN-*deficient GBMs.

## Discussion

Here, we presented a systematic method for identification and validation of putative tumor suppressors in GBM through selection of candidate genes based on genomic analysis, in vivo loss-of-function screen, and subsequent validation in patient-derived xenograft models. Furthermore, we have previously established such approaches in GBM and identified Nemo-like kinase as a negative regulator of mesenchymal activity in GBM (Sa et al., 2015). Our screening approach allowed us to identify PIP4K2A as a putative tumor suppressor gene and a key regulator of GBM pathogenesis. We showed that PIP4K2A suppresses human GBM growth in vitro and in vivo, stem-like characteristics, and PI3K/AKT signaling through Cbl-mediated p85/p110 PI3K complex degradation in *PTEN*-deficient tumors.

PIP4K2A gene encodes for phosphatidylinositol-5-phosphate 4-kinase, type II, α (PtdIns5P 4-kinase α). It belongs to the class II PIPK family, also known as the phosphatidylinositol-5-phosphate 4-kinase family, and the major function of these proteins is to phosphorylate at position four of the inositol ring to generate a new lipid messenger, the phosphatidylinositol-4,5-bisphosphate (PtdIns4,5P2). PtdIns4,5P2 plays a vital role in phosphoinositide signaling, regulating several essential cellular processes, including vesicle transport, cellular proliferation, adhesion, apoptosis, and nuclear events (McCrea and De Camilli, 2009). A full detailed function of PIP4K2A protein under cellular mechanism still remains elusive, and recent findings suggest that its family is actively involved in oxidative stress, cellular senescence, and tumor growth (Emerling et al., 2013; Fiume et al., 2015).

Down-regulation of PIP4K2A attenuates growth of acute leukemia cells but not of primary normal human hematopoietic stem and progenitor cells. Whether tumor suppression is dependent only on the loss of PIP4K activity remains unclear (Fiume et al., 2015). Furthermore, reasons behind down-regulation of PIP4K2A only affecting the tumor cells, leaving the growth of normal hematopoietic stem and progenitor cells intact, also remain unexplored (Fiume et al., 2015; Jude et al., 2015). Moreover, aberration of PIP4K2A and PIP4K2B are frequently observed in solid tumors, including breast carcinomas and lung adenocarcinomas (Fig. S4; Emerling et al., 2013; Keune et al., 2013; Jude et al., 2015), regulating genes that are involved in cell cycle progression, epithelial–mesenchymal transition, and reactive oxygen accumulation and metabolism, ultimately affecting tumor growth. However, a detailed description of PIP4K and PI5P directly influencing specific gene transcription remains elusive. Additionally, Jones et al. (2013) previously demonstrated that induction of oxidative stress inhibits PIP4K2A activity, and PIP4K2A overexpression reduces clonogenic growth. In contrast, PIP4K2A increased cell viability in response to oxidative stress in U2OS cells, osteosarcoma cell line.

There are three mammalian isoforms of PtdIns5P 4-kinases type II: α, β, and γ (Clarke et al., 2007). PIP4K2A is located in both the cytoplasm and nucleus and forms homodimer or heterodimer with PtdIns5P 4-kinase β or γ (Bultsma et al., 2010; Clarke et al., 2010). In vitro assays indicated that PIP4K2A has the highest enzyme activity compared with the other isoforms (Clarke and Irvine, 2012). To examine whether the tumor-suppressive role of PIP4K2A is dependent on its kinase activity, we generated and integrated inactive mutants PIP4K2A^N251S^ (Fedorenko et al., 2008) and PIP4K2A^G131L/Y138F^ into GBM cells and assessed their inhibition effects on AKT phosphorylation and short-term proliferative kinetics. Both inactive mutants PIP4K2A^N251S^ and PIP4K2A^G131L/Y138F^ showed similar inhibition rate to that of wild-type *PIP4K2A* (Fig. S5). These results suggest that kinase activity of PIP4K2A is not essential in initiating p85/p110 PI3K complex degradation in GBM.

Class IA PI3Ks are heterodimeric enzymes composed of p85 family regulatory subunits (p85α, p85β, and p55γ) and p110 family catalytic subunits (p110α, p110β, and p110δ). The interaction between p85 and p110 is critical for the stability of p110 (Yu et al., 1998a). Moreover, an excess level of p85 to p110 initiates competition for the binding of p85/p110 complex to Tyr-phosphorylated activators such as IRS1 (Luo et al., 2005) and could contribute to PTEN activation, shutting off its signaling via the PTEN-associated complex (Rabinovsky et al., 2009; Chagpar et al., 2010). *PTEN* mutation or deletion, often through the complete loss of its locus on chromosome 10q, is found largely in GBM patients (Ali-Osman, 2005; Cancer Genome Atlas Research Network, 2008; Parsons et al., 2008). Furthermore, the genomic loss of *PTEN* dramatically enhances gliomagenesis in a number of murine model systems (Hu et al., 2005; Wei et al., 2006). We found that GBM patients with *PIP4K2A* wild-type exhibited significantly favorable survival outcomes compared with the patients with *PIP4K2A* deletion in *PTEN*-deleted cohorts, which suggests clinical relativity (Fig. 6, L and M). We suspected that the growth inhibition effect of PIP4K2A could be associated with the presence of PTEN and p85.

In the present study, we demonstrate in vivo RNAi screen as a powerful tool to identify and validate putative tumor suppressors in GBM. The PI3K signaling pathway is frequently dysregulated in GBM. Given the profound and unprecedented effects of PIP4K2A on tumor growth inhibition in vitro and in vivo through regulating PI3K signaling via p85/p110 component degradation, we highlight the newly discovered role of PIP4K2A in GBM.

## Materials and methods

### In vivo RNAi screen

shRNAs were obtained from Open Biosystems as pGIPZ lentiviral vectors. The overall shRNA barcode-screening procedure was performed as described previously (Sa et al., 2015). For the shRNA screening procedure, different sets of cells were infected with a pool of ∼200 lentiviral shRNAs targeting 24 human genes at a representation of ∼500 cells per shRNAs at a multiplicity of infection of 1. On day 2 after the infection, puromycin (1 µg/ml) was added to eliminate any non-transduced cells, and the selection procedure proceeded for the next 3 d. Afterward, 100,000 remaining cells were injected into the recipient mice, and the control populations were harvested. For each corresponding samples, shRNA barcodes were PCR-amplified from genomic samples and analyzed through deep sequencing technology (Illumina High-Seq 2000). Each shRNA read was normalized to its respective whole population, and changes in the relative abundance of each shRNA in the library were measured.

### GBM patient-derived specimens and primary cell culture

After receiving informed consent, GBM specimens were obtained from patients undergoing surgery at the Samsung Medical Center in accordance with the Samsung Medical Center Institutional Review Board. This work was performed in compliance with all relevant ethical regulations for research using human specimens. Patient-derived primary GBM cells and normal NPCs were cultured as previously described (Lee et al., 2006; Son et al., 2009; Kim et al., 2013). For sphere culture, GSCs and NPCs were cultured in the Neurobasal medium under neurosphere culture condition (Lee et al., 2006; Son et al., 2009; Kim et al., 2013). Normal NPCs (Lonza; PT-2599) were purchased and cultured as recommended.

### Plasmids and lentiviral transduction

Lentiviral vectors expressing shRNAs for PIP4K2A, PI3K (p85), and Cbl were purchased from Sigma-Aldrich: PIP4K2A (#1, TRCN0000356569; #2, TRCN0000356567; #3, TRCN0000356495; #4, TRCN0000356493; #5, TRCN0000006009), p85 (#1, TRCN0000039904; #2, TRCN0000039906; #3, TRCN0000039907; #4, TRCN0000039563), and Cbl (#1, TRCN0000039723; #2, TRCN0000039724; #3, TRCN0000039726; #4, TRCN0000039727; #5, TRCN0000039725). The following shRNAs were used throughout the experiments: PIP4K2A (all five shRNAs were used in the in vivo RNAi screen; shRNAs #2, #3, and #4 for the in vivo RNAi validation experiment; and shRNA #3 for the in vitro cellular growth and immunoblot experiments), p85 (shRNA #1 was used for all in vitro experiments), and Cbl (shRNA #1 was used for all in vitro experiments). To generate the recombinant lentivirus that expresses PIP4K2A wild-type and PI3KC-E545K mutant, a cloning package including entry (pCR8/GW/TOPO Vector) and lentiviral destination (pLenti6/V5-Dest) vectors were used (Invitrogen) and validated by sequencing and immunoblot analysis. As V5 epitope consists of 15 amino acids, and its molecular mass is measured at ∼5 kD, exogenous PIP4K2A protein shows higher molecular mass compared with the endogenous PIP4K2A. For viral production, 293T cells were cotransfected with a lentiviral expression vector and packaging plasmid (psPAX2 and pCMV-VSVG) using CalPhos Mammalian Transfection Kit (Clontech). Virus-containing supernatants were collected and concentrated by ultracentrifugation. The titer of each lentivirus was determined by serial dilution.

### Orthotopic GBM xenograft models

All mouse experiments were performed according to the guidelines of the Animal Use and Care Committees at the Samsung Medical Center and Association for Assessment and Accreditation of Laboratory Animal Care–accredited guidelines (Kim et al., 2013; Sa et al., 2015). 6-wk-old female BALB/c nude mice (Orient Bio) were used for intracranial transplantation. Patient-derived glioma cells (1 × 10^5^ per mouse) were injected into the brains of mice by stereotactic intracranial injection (coordinates: 2 mm anterior, 2 mm lateral, 2.5 mm depth from the dura). Mice were sacrificed either when 25% body weight loss or neurological symptoms (lethargy, ataxia, and seizures) were observed.

For in vivo growth competition assays, GBM cells were labeled with either GFP or RFP using lentiviral infection. After checking fluorescence signals through FACS analysis, GFP-labeled GBMs were infected with control lentivirus (empty control vector), and RFP-labeled GBMs were infected with PIP4K2A-expressing lentivirus. GBM samples were dissociated into single cells using Accutase, and 10^5^ GBM cells were mixed with 5 µl HBSS buffer and injected intracranially into the striatum of adult nude mice by using a stereotactic device (Kopf Instruments). Mice with tumor formation were sacrificed for primary culture. Primary tumors were harvested, minced, and incubated in CDD1 for 10 min. Dissociated cells were filtered through 40-µm mesh and then processed for FACS analysis using FACS Calibur flow cytometry (BD Biosciences). The expression levels of GFP and RFP were determined using the FlowJo program.

### Cell proliferation assay, cell counting, and LDAs

Cell proliferation was measured using the EZ-Cytox cell viability kit (DAEIL Lab) according to the manufacturer’s protocol. GBM cells were transduced with either control lentivirus (empty control vector) or PIP4K2A-expressing lentivirus. PIP4K2A and control cells were plated onto 96-well plates at 10^3^ cells per well, and each sample was plated and incubated in quintuplicate for 6 d. Cell proliferation was detected with the Ez-Cytox according to the manufacturer’s instructions. Optical density values were measured by using a microplate reader at an absorbance of 450 nm. For a direct cell count, the number of viable cells was counted by the trypan blue dye exclusion assay with a hemocytometer.

For LDAs, cells were transduced with either control lentivirus (empty vector) or PIP4K2A-expressing lentivirus. PIP4K2A or control infected cells were plated in 96-well plates at a range of 1–100 cells per well. After 1–2 wk, the number of wells without spheres was counted. At the time of quantification, each well was examined for the formation of tumorspheres. The LDA clonogenic index is calculated as the inverse of the x-intercept of the regression between the number of wells without spheres (y axis) and the number of cells seeded at a range of 1–100 cells per well (x axis; Kim et al., 2013; Jin et al., 2017; Yin et al., 2017). Stem cell frequency was calculated by using extreme limiting dilution analysis (http://bioinf.wehi.edu.au/software/elda/).

### TMA and tumor samples

For analysis of PIP4K2A, PI3K (p85), and PI3K (p110) by immunohistochemistry, TMA containing neoplastic and matching non-neoplastic tissues was used (Sa et al., 2015). Brain tissue samples were fixed by formalin and embedded in paraffin; then sections of paraffin-embedded glioma specimens were stained with human antibodies against PIP4K2A, p85, or p110.

### Antibodies

The following antibodies were used for immunoblotting: PIP4K2A 1:1,000 (AP8041b; Abgent), p85 1:1,000 (4257S; Cell Signaling), p110α 1:1,000 (4249S; Cell Signaling), p110β 1:1,000 (3011P; Cell Signaling), pAKT 1:1,000 (4060S; Cell Signaling), AKT 1:1,000 (4691S; Cell Signaling), pS6K 1:1,000 (9234S; Cell Signaling), S6K 1:1,000 (2708S; Cell Signaling), Cbl 1:1,000 (2747S; Cell Signaling), PTEN 1:1,000 (9188S; Cell Signaling), and β-actin 1:5,000 (A5316; Sigma-Aldrich). The following antibodies were used for immunohistochemistry or immunofluorescence: PIP4K2A 1:50 (AP8041b; Abgent), pAKT 1:200 (4060S; Cell Signaling), p85 1:100 (ab86714; Abcam), and p110 1:100 (ab1678; Abcam).

### Immunostaining analysis

Cells and tissues were fixed in 4% PFA for 20 min. After blocking and permeabilizing with 0.3% Triton X-100 and 10% goat or donkey serum in PBS for 1 h, samples were probed with the following primary antibodies for overnight at 4°C: PIP4K2A, p85, and pAKT. Appropriate fluorescence-tagged secondary antibodies (Invitrogen) and 4,6-diamidino-2-phenylindole dihydrochloride (Roche) were used for visualization. Microscopy was done with confocal microscope imaging.

### Co-IP and immunoblot assays

Protein co-IP in GBM cells was performed essentially as previously reported (Kim et al., 2013). GBM cells were lysed in Pierce IP non-denaturing lysis buffer (25 mM Tris-HCl pH 7.4, 150 mM NaCl, 1% NP-40, 1 mM EDTA, 5% glycerol; Thermo Scientific Pierce; 8778) supplemented with proteinase inhibitor and phosphatase inhibitor. For IP, 500 µg of proteins was isolated from GBM cells, incubated for overnight with 2 µg of primary antibody or IgG (Santa Cruz; SC-2025 or SC-2027), conjugated to protein A/G beads (Santa Cruz; SC-2003), washed three times using Pierce IP lysis buffer, and then separated on SDS-PAGE gels. For immunoblotting, blots were incubated with PIP4K2A, p85, p110α, p110β, pAKT, AKT, pS6K, S6K, Cbl, PTEN, Ub, or β-actin overnight at 4°C. After washing with Tris-buffered saline with Tween 20, the blots were incubated with HRP-conjugated secondary antibody (in case of a co-IP sample: light-chain HRP secondary antibody) for 1 h at room temperature. Detection was performed by using the SuperSignal West Pico Chemiluminescent Substrate (ECL; Thermo Scientific Pierce).

### Statistical analysis

All data were expressed as means ± SD or ± SEM from at least three independent experiments. Quantification in immunostaining analyses was performed by using ImageJ software (National Institutes of Health), and results were presented as the percentage of pixels mean area. For the animal survival studies, P values were determined by log-rank test. Student’s *t* test was used to determine statistical significance. P values <0.05 were considered significant.

### Online supplemental material

Fig. S1 shows correlation between *PIP4K2A* and *PTEN* expressions and Kaplan–Meier survival analysis of GBM patients based on *PIP4K2A* expression. Fig. S2 shows the effects of overexpression and shRNA-mediated knockdown of PIP4K2A on cellular growth and PIP4K2A, p85, and pAKT protein expression levels. Fig. S3 shows effects of PIP4K2A or PTEN overexpression with or without MG132 treatment on PIP4K2A, PTEN, p85, pAKT, or ubiquitin protein expression levels or binding activities in GBMs or NPCs. Fig. S4 shows *PIP4K2A* mRNA expression levels in breast carcinoma versus normal breast tissues (left) and lung adenocarcinoma versus normal lung tissues (right). Fig. S5 shows the effects of PIP4K2A kinase mutants on PIP4K2A and p85 protein expression levels and cellular growth.

## Supplementary Material

Supplemental Materials (PDF)
