# Supplementary material for: PIP4K2A as a negative regulator of PI3K in PTEN-deficient glioblastoma
Source: J Exp Med. 2019 Mar 21;216(5):1120–34. doi: 10.1084/jem.20172170 (PMC6504209; doi:10.1084/jem.20172170)
Supplement: Supplemental Materials (PDF) [file JEM_20172170_sm.pdf]

Supplemental material

Shin et al., https://doi.org/10.1084/jem.20172170

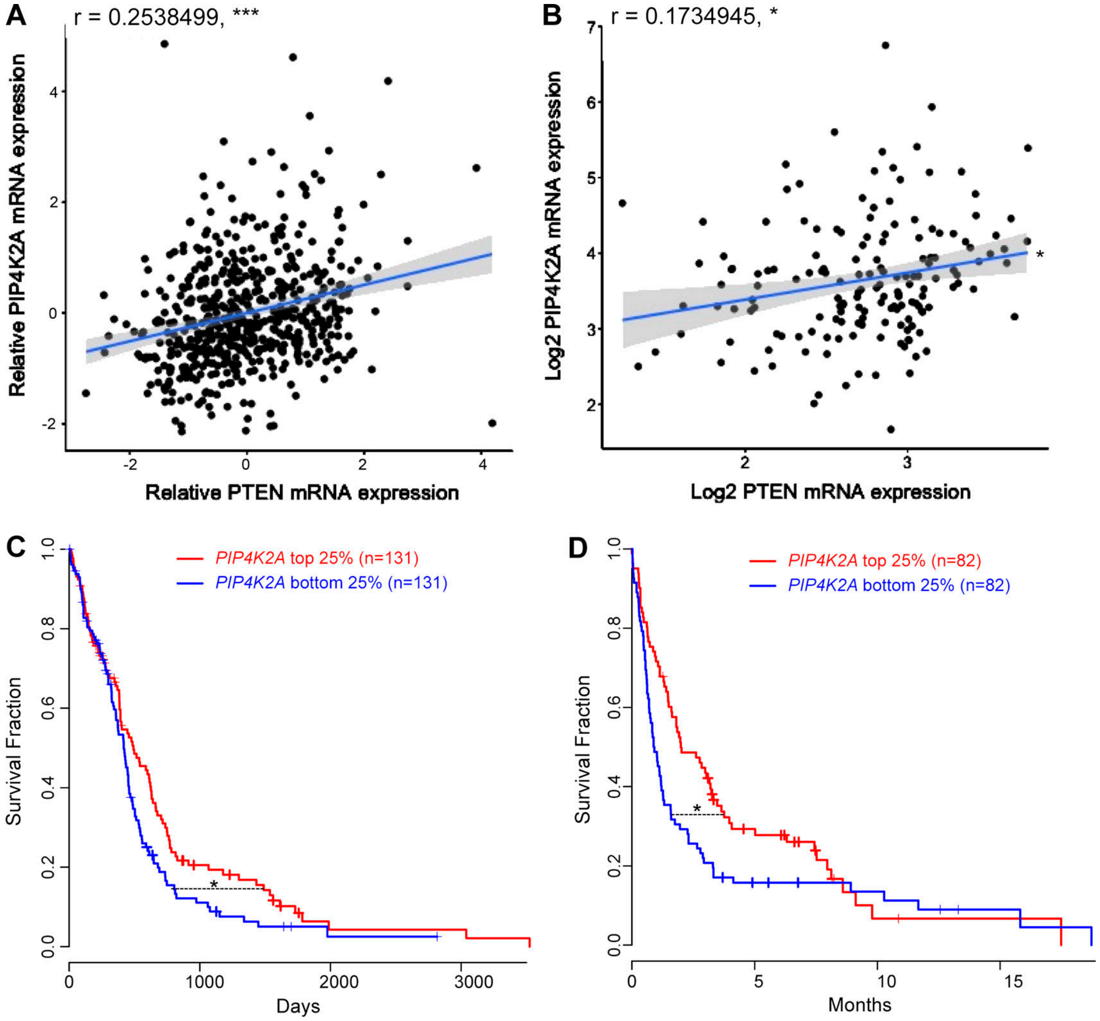

Figure S1. **Transcriptome significance of PIP4K2A in GBM. (A and B)** Scatter plot showing linear correlation between *PIP4K2A* and *PTEN* mRNA expression levels in TCGA U133A microarray (left) and RNA sequencing (right) datasets. The P values were obtained using the Pearson correlation test. **(C and D)** Kaplan–Meier survival curve analysis of GBM patients based on the *PIP4K2A* mRNA expression level in TCGA (left) and Gravendeel et al. (2009) datasets (right). The P values were obtained using the two-sided log-rank test. *, P ≤ 0.05; ***, P ≤ 0.001.

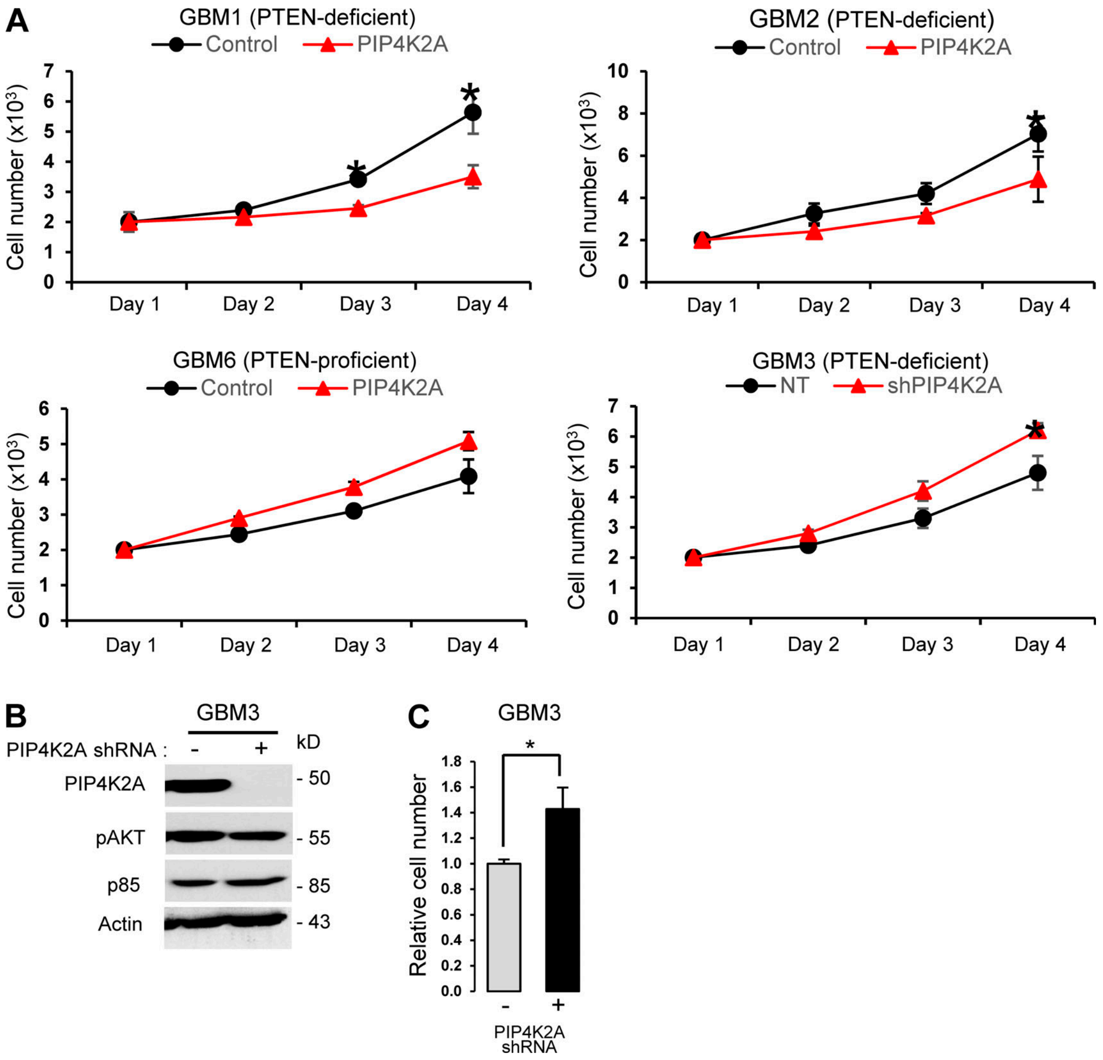

Figure S2. **Effects of PIP4K2A in cellular growth. (A)** Effects of PIP4K2A overexpression or shRNA-mediated knockdown on cellular proliferation. The P values were calculated using the two-tailed Student's *t* test. **(B)** Immunoblot analysis of PIP4K2A, pAKT, and p85 in GBM cells that were transduced with either control or PIP4K2A shRNA. Actin was used as a loading control. **(C)** Proliferation assay of GBM cells from B. The P values were calculated using the two-tailed Student's *t* test. Values are presented as mean ± SD (n = 4). Data shown in A are representative of two independent and reproducible experiments. Data shown in B and C are representative of three independent and reproducible experiments. *, P ≤ 0.05.

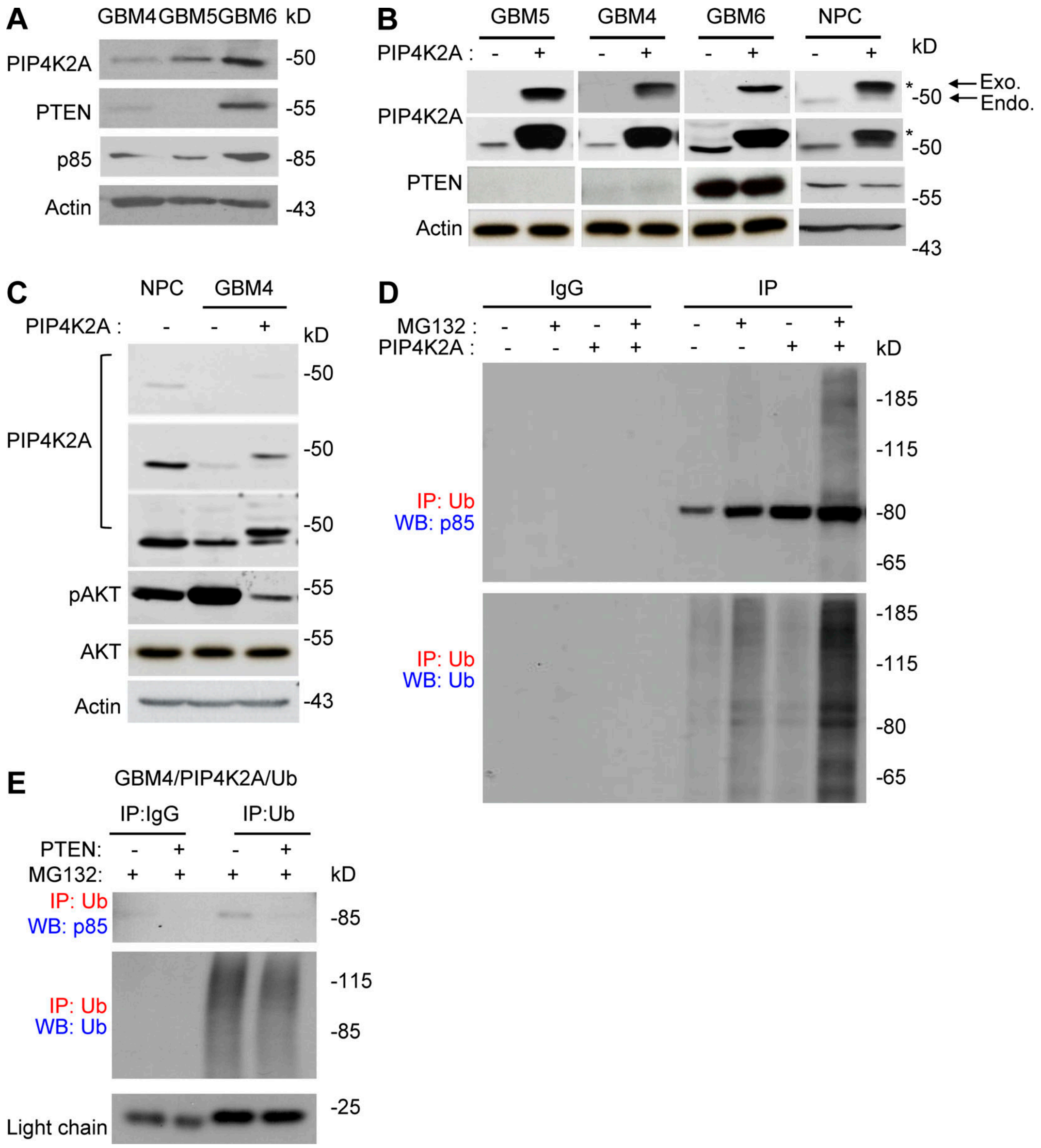

Figure S3. **Effects of PIP4K2A and PTEN in PI3K signaling pathway. (A)** Immunoblot analysis of PIP4K2A, PTEN, and p85 in GBM cells. Actin was used as a loading control. **(B)** Immunoblot analysis of PIP4K2A and PTEN in GBM cells and NPCs that were transduced with either control or PIP4K2A. Actin was used as a loading control. **(C)** Immunoblot analysis of PIP4K2A, pAKT, and AKT in NPCs and GBMs that were transduced with either control of PIP4K2A. Actin was used as a loading control. **(D)** Co-IP of ubiquitin and p85 in GBM cells that were transduced with either PIP4K2A or control and treated with or without MG132. IgG represents a control antibody used for IPs. **(E)** Co-IP of ubiquitin and p85 in GBM cells that were transduced with either PTEN or control and treated with MG132. IgG represents a control antibody used for IPs. Data shown in A–E are representative of three independent and reproducible experiments. Endo., endogenous; Exo., exogenous; WB, Western blot.

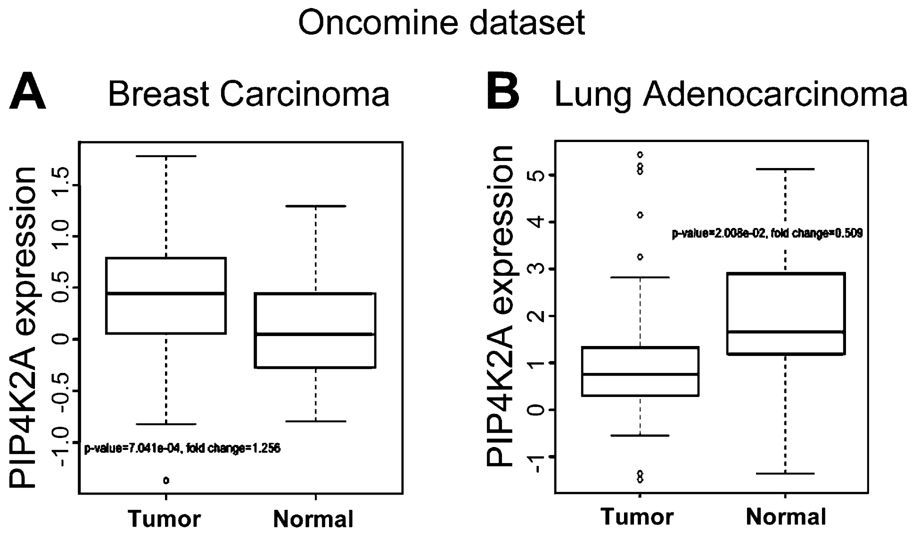

Figure S4. **Clinical relevance of PIP4K2A in breast and lung cancers. (A)** Oncomine microarray data analysis for PIP4K2A expression in breast carcinoma versus normal breast tissues (P < 0.0007). **(B)** Oncomine microarray data analysis for PIP4K2A expression in lung adenocarcinoma versus normal lung tissues (P < 0.02). The P values were calculated by using the two-sided Wilcoxon rank sum test.

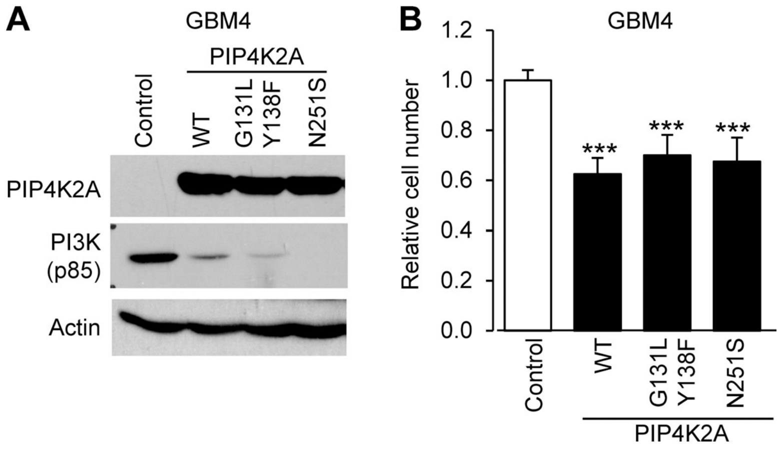

Figure S5. **Effects of PIP4K2A-kinase mutations in GBM. (A)** Immunoblot analysis of PIP4K2A and p85 in GBM cells that were transduced with control, PIP4K2A WT, PIP4K2A G131L mutation, PIP4K2A Y138F mutation, or PIP4K2A N251S mutation. Actin was used as a loading control. **(B)** Proliferation assay of GBM cells from A. Values are presented as mean ± SD (n = 5). The P values were calculated using the two-tailed Student's t test. ***, P ≤ 0.001. Data shown in Figure A and B are representative of three independent and reproducible experiments.

References

Gravendeel, L.A., M.C. Kouwenhoven, O. Gevaert, J.J. de Rooi, A.P. Stubbs, J.E. Duijm, A. Daemen, F.E. Bleeker, L.B. Bralten, N.K. Kloosterhof, et al. 2009. Intrinsic gene expression profiles of gliomas are a better predictor of survival than histology. *Cancer Res.* 69:9065–9072. https://doi.org/10.1158/0008-5472.CAN-09-2307
